# Berberine Attenuates Myocardial Ischemia/Reperfusion Injury by Reducing Oxidative Stress and Inflammation Response: Role of Silent Information Regulator 1

**DOI:** 10.1155/2016/1689602

**Published:** 2015-12-14

**Authors:** Liming Yu, Qing Li, Bo Yu, Yang Yang, Zhenxiao Jin, Weixun Duan, Guolong Zhao, Mengen Zhai, Lijun Liu, Dinghua Yi, Min Chen, Shiqiang Yu

**Affiliations:** ^1^Department of Cardiovascular Surgery, Xijing Hospital, Fourth Military Medical University, Xi'an 710032, China; ^2^Beijing Aortic Disease Center, Anzhen Hospital, Capital Medical University, Beijing 100029, China; ^3^Department of Biomedical Engineering, Fourth Military Medical University, Xi'an 710032, China; ^4^School of Clinical Medicine, Ningxia Medical University, Yinchuan 750004, China; ^5^College of Life Science, Northwest University, Xi'an 710069, China; ^6^Department of Anesthesiology, Xijing Hospital, Fourth Military Medical University, Xi'an 710032, China

## Abstract

Berberine (BBR) exerts potential protective effect against myocardial ischemia/reperfusion (MI/R) injury. Activation of silent information regulator 1 (SIRT1) signaling attenuates MI/R injury by reducing oxidative damage and inflammation response. This study investigated the antioxidative and anti-inflammatory effects of BBR treatment in MI/R condition and elucidated its potential mechanisms. Sprague-Dawley rats were treated with BBR in the absence or presence of the SIRT1 inhibitor sirtinol (Stnl) and then subjected to MI/R injury. BBR conferred cardioprotective effects by improving postischemic cardiac function, decreasing infarct size, reducing apoptotic index, diminishing serum creatine kinase and lactate dehydrogenase levels, upregulating SIRT1, Bcl-2 expressions, and downregulating Bax and caspase-3 expressions. Stnl attenuated these effects by inhibiting SIRT1 signaling. BBR treatment also reduced myocardium superoxide generation, gp91^phox^ expression, malondialdehyde (MDA) level, and cardiac inflammatory markers and increased myocardium superoxide dismutase (SOD) level. However, these effects were also inhibited by Stnl. Consistently, BBR conferred similar antioxidative and anti-inflammatory effects against simulated ischemia reperfusion injury in cultured H9C2 cardiomyocytes. SIRT1 siRNA administration also abolished these effects. In summary, our results demonstrate that BBR significantly improves post-MI/R cardiac function recovery and reduces infarct size against MI/R injury possibly due to its strong antioxidative and anti-inflammatory activity. Additionally, SIRT1 signaling plays a key role in this process.

## 1. Introduction

Myocardial ischemia/reperfusion injury (MI/RI) is the primary cause of cardiac failure as well as morbidity and mortality after myocardial infarction [1–3]. Accumulating experimental evidence demonstrates that the development of oxidative stress induced by the generation of reactive oxygen species (ROS) during the acute reperfusion phase plays a pivotal role in the etiopathogenesis of MI/RI [3, 4]. Reactive oxygen species trigger subsequent leukocyte chemotaxis and inflammation, thus causing severe cardiac damage [4, 5]. Therefore, protecting cardiomyocyte from ROS damage could be a rational method for ameliorating MI/RI.

**Figure 1:**
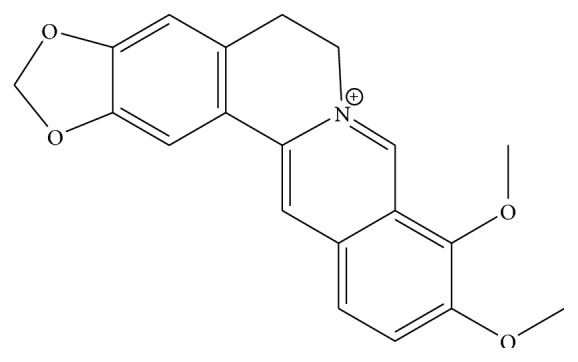
Molecular structure of berberine.

Berberine (BBR, Figure 1) is a natural isoquinoline alkaloid isolated from the Chinese herb* Rhizoma coptidis*, which has been widely used in Chinese herbal medicine [6]. In recent years, BBR has gained much attention owing to its multiple biochemical and pharmacological activities, including antiviral, anticancer, and cardioprotective effects [7–10]. Previous experimental data also indicated that BBR might be utilized as a suitable antioxidative agent in a series of pathological conditions [11–13]. Several studies have also revealed that certain BBR derivatives exert cardioprotective effect by reducing oxidative damage [14, 15]. However, few studies reported the antioxidative and anti-inflammatory effects of BBR in MI/R condition.

Silent information regulator 1 (SIRT1) is a nicotinamide adenine dinucleotide- (NAD-) dependent histone deacetylase that regulates a variety of important metabolic and physiologic processes including stress resistance, metabolism, apoptosis, and energy balance [16, 17]. Previous studies have demonstrated that SIRT1 deacetylates and activates forkhead box O (FOXO), which synthesizes antioxidants, such as manganese superoxide dismutase (MnSOD) and catalase, thereby reducing oxidative damage [18, 19]. In addition, it has been revealed that BBR may confer protective effect via activating SIRT1 signaling in a variety of pathological conditions [20, 21]. However, whether SIRT1 signaling plays a role in BBR's antioxidative and anti-inflammatory effects against MI/RI and its underlying mechanisms are still unknown.

The aims of the present study were to (1) examine whether BBR treatment confers antioxidative and anti-inflammatory effects in MI/R condition and (2) investigate the role of SIRT1 signaling in BBR's cardioprotective effect, especially in its antioxidative activity.

## 2. Materials and Methods

### 2.1. Materials

BBR, Evans blue (EB), triphenyltetrazolium chloride (TTC), 4′,6-diamino-2-phenylindole (DAPI), and protease inhibitor cocktail were purchased from Sigma-Aldrich (St. Louis, MO, USA). Sodium carboxymethylcellulose (CMC-Na) was purchased from Solarbio Technology (Beijing, China). Lactate dehydrogenase (LDH) assay kit, creatine kinase (CK), malondialdehyde (MDA), superoxide dismutase (SOD), superoxide generation, IL-6, and myeloperoxidase (MPO) assay kits were purchased from Jiancheng Bioengineering Institute (Nanjing, China). TNF-*α* ELISA kit was purchased from R&D Corporation (Minneapolis, USA). Terminal deoxynucleotidyl transferase-mediated dUTP nick end labeling (TUNEL) assay kit was obtained from Roche Molecular Biochemicals (Mannheim, Germany). BCA protein quantification kit was purchased from Merck Millipore Technology (Darmstadt, Germany). The primary antibodies against SIRT1, Ac-Foxo1, gp91^phox^, caspase-3, Bcl-2, Bax, *β*-actin, and SIRT1 siRNA were purchased from Santa Cruz Biotechnology (CA, USA). Rabbit anti-goat, goat anti-rabbit, and goat anti-mouse secondary antibodies were purchased from the Zhongshan Company (Beijing, China).

### 2.2. Animals

Male Sprague-Dawley rats (7–9 weeks) weighing 250–300 g were purchased from the Center of Experimental Animal in the Fourth Military Medical University, China. All animals used in this study were cared for in accordance with the Guide for the Care and Use of Laboratory Animals published by the United States National Institute of Health (NIH publication number 85-23, revised 1996), and all procedures were approved by the Committee of Experimental Animals of the Fourth Military Medical University.

### 2.3. Myocardial Ischemia/Reperfusion Protocol

Myocardial ischemia/reperfusion operation was carried out as described before [22]. Briefly, rats were anesthetized by 3% pentobarbital sodium. Myocardial ischemia was carried out by exteriorizing the heart through a left thoracic incision, placing a 6-0 silk suture and making a slipknot around the left anterior descending coronary artery. After 30 min of ischemia, the slipknot was released and the myocardium was reperfused for 4 hr (for analysis of protein expression), 6 hr (for quantification of myocardial apoptosis and infarct size), and 24 hr (for cardiac function determination). Sham group underwent the same operation procedures except that the suture passed under the left coronary artery was left untied. Before and during the surgery, animals received different treatment.

### 2.4. Simulated Ischemia/Reperfusion Treatment

H9C2 embryonic rat myocardium-derived cells (Shanghai Tiancheng Technology Co., Ltd.), a well-characterized and widely used cell line to study myocardial cell ischemia, were cultured in DMEM supplemented with 10% heat-inactivated fetal bovine serum. The SIR treatment was performed using physiological concentrations of potassium, hydrogen, and lactate. The procedure was performed as described previously [23]. Briefly, cardiomyocytes were exposed to an ischemic buffer containing (in mmol/L) 137 NaCl, 12 KCl, 0.49 MgCl_2_, 0.9 CaCl_2_, and 4 HEPES. This buffer was also supplemented with (in mmol/L) 10 deoxyglucose, 0.75 sodium dithionate, and 20 lactate. The buffer pH was 6.5. Cardiomyocytes were incubated for 2 hr in a humidified cell culture incubator (21% O_2_, 5% CO_2_, and 37°C). Reperfusion was performed by returning the cells to normal culture medium for 4 hr in a humidified cell culture incubator (21% O_2_, 5% CO_2_, and 37°C).

### 2.5. Experimental Group

Step 1 was carried out to determine the role of SIRT1 signaling in BBR's cardioprotective effect in animal model. SD rats were randomly divided into the following experimental groups (*n* = 40). In Group 1 (Sham), normal rats received no treatment but Sham operation. In Group 2 (MI/R + V), normal rats received vehicle treatment (0.5% CMC-Na solution 2 mL per day by oral gavage for 2 weeks) and then were subjected to MI/R operation. In Group 3 (MI/R + BBR), rats were given BBR by oral gavage (dissolved in 0.5% CMC-Na solution) at a dose of 200 mg/kg/d for 2 weeks and then subjected to MI/R operation. In Group 4 (MI/R + BBR + Stnl), rats were treated with BBR as well as Stnl (2 mg/kg/d for 1 week before MI/R operation by intraperitoneal injection) and then subjected to MI/R operation. To evaluate the effect of BBR or Stnl treatment on the heart function of Sham operated rats, we also treated SD rats with BBR or Stnl, respectively. Then, rats were subjected to Sham operation. The dosages of BBR and Stnl in vivo were chosen based on previous studies [24, 25].

Step 2 was designed to investigate the role of SIRT1 signaling in BBR's protective action in H9C2 cardiomyocytes. The dosage of BBR in vitro was chosen based on the previous experiments [24]. Furthermore, we tested the toxic effect of BBR treatment at 0.5, 5, 50, and 500 *μ*mol/L for 8 hr on H9C2 cell viability in vitro. The dose of 50 *μ*mol/L BBR was employed in our study. BBR stock solution was prepared and diluted with DMEM. After preparation, the cardiomyocytes were randomly divided into the following experimental groups (*n* = 8). In Group 1 (control siRNA), the cardiomyocytes were transfected with control siRNA, strictly following the manufacturer's instructions. After the transfection procedure was completed, the cardiomyocytes were incubated in DMEM with the transfection mixture for 24 hr and then incubated in normal DMEM for 16 hr. In Group 2 (SIR + control siRNA), the cardiomyocytes were transfected with control siRNA following the same routine. After the transfection and the incubation in DMEM with the transfection mixture were completed, cells were also incubated in normal DMEM for 10 hr and then subjected to SIR. In Group 3 (SIR + BBR + control siRNA), the cardiomyocytes were transfected with control siRNA following the same routine. After the transfection and the incubation were completed, cells were incubated in normal DMEM for 2 hr and then treated with BBR (50 *μ*mol/L) for 8 hr. Then, the cells were subjected to SIR. In Group 4 (SIR + BBR + SIRT1 siRNA), the cardiomyocytes were transfected with SIRT1 siRNA, strictly following the manufacturer's instructions. After the transfection procedure was completed, the cardiomyocytes were incubated in DMEM with the transfection mixture for 24 hr, incubated in normal DMEM for 2 hr, and then treated with BBR (50 *μ*mol/L) for 8 hr. Then, the cells were subjected to SIR. The cells were harvested after the SIR treatment for further analysis.

### 2.6. Echocardiography

First, rats were anesthetized with isoflurane 24 hr after MI/R operation. Then, two-dimensional and M-mode echocardiographic measurement was carried out with a VEVO 770 high-resolution in vivo imaging system (Visual Sonics, Toronto, Canada). Left ventricular ejection fraction (LVEF) and left ventricular fractional shortening (LVFS) were measured as described by our previous study [22].

### 2.7. Determination of Myocardial Infarction and Apoptosis

Myocardial infarct size was determined by means of EB-TTC staining and a digital imaging system (infarct area/area-at-risk × 100%) after 6 hr of reperfusion [22]. Myocardial apoptosis was analyzed by TUNEL assay using an in situ cell death detection kit as described before [22]. The index of apoptosis was expressed by number of apoptotic cardiomyocytes/the total number of cardiomyocytes counted × 100%.

### 2.8. Determination of Serum CK and LDH

Blood samples (0.5 mL) were drawn after 6 hr of reperfusion. Serum CK and LDH levels were determined spectrophotometrically (Beckman DU 640, Fullerton, CA) according to the manufacturer's instructions [22].

### 2.9. Quantification of Superoxide Production

Superoxide production in tissues and cells was measured by lucigenin-enhanced chemiluminescence as described previously [26]. Superoxide production was expressed as relative light units (RLU) per second per milligram heart weight (RLU/mg/s).

### 2.10. Determination of Tissue Malondialdehyde and Superoxide Dismutase

The MDA level and activities of antioxidant SOD in heart homogenates were determined spectrophotometrically as previously described [26].

### 2.11. Detection of IL-6 and TNF-*α* Level

After reperfusion, the levels of IL-6, TNF-*α* in myocardial tissue homogenate, cardiomyocytes supernatant, and serum were detected in strict accordance with manufacturer's instructions [27, 28]. BCA kit was used to detect the protein quantization.

### 2.12. Determination of Myeloperoxidase (MPO) Level

After reperfusion, the myocardial tissue was placed at −70°C for preservation. MPO test kit was used to detect level of MPO in the myocardial tissue according to manufacturer's instructions [28].

### 2.13. Cell Viability Analysis

H9C2 cardiomyocytes were seeded in 96-well culture plates. After different treatment, the SIR was performed. Cell viability was measured by 3-(4,5-dimethylthiazol-2-yl)-2,5-diphenyltetrazolium bromide (MTT) assay as described before [23]. Briefly, after the cells were treated and washed with PBS, 10 *μ*L of MTT dye was added to each well at a final concentration of 0.5 mg/mL. After 4 hr of incubation, 100 *μ*L of DMSO was added to dissolve the formazan crystals, and the absorbance was measured using a microtiter plate reader (SpectraMax 190, Molecular Device, USA) at a wavelength of 490 nm. The cell viability was calculated by dividing the optical density of samples by the optical density of control group.

### 2.14. Determination of Cellular Apoptosis

After the treatment, cardiomyocytes were fixed in paraformaldehyde (4%) for 24 hr. Cellular apoptosis was also analyzed by performing a TUNEL assay using the in situ cell death detection kit according to the manufacturer's instructions [23]. The apoptotic index was expressed as the number of positively stained apoptotic cardiomyocytes/the total number of cardiomyocytes counted × 100%.

### 2.15. Western Blotting

The myocardium and cardiomyocytes samples were lysed in lysis buffer on ice for 20 min, and the lysates were clarified by centrifugation at 4°C for 15 min at 12,000 rpm. After quantitation of protein concentration with BCA protein assay kit, 30 *μ*g of total protein was separated by SDS-PAGE and then transferred to a polyvinylidene difluoride membrane (Millipore, USA). The membranes were blocked for 4 hr at 37°C with 5% nonfat dry milk and then incubated with primary antibody including SIRT1, Ac-Foxo1, gp91^phox^, caspase-3, Bcl-2, Bax, and *β*-actin (1 : 500 in TBST) over night at 4°C. After three washings with TBST, the membranes were incubated with secondary antibody in TBST solution for 30 min at 37°C and then washed as above. The positive protein bands were developed using a chemiluminescent system, and the bands were scanned and quantified by densitometric analysis using an image analyzer Quantity One System (Bio-Rad, Richmond, CA, USA).

### 2.16. Statistical Analysis

All values are presented as mean ± SEM. Differences were compared by ANOVA followed by Bonferroni correction for post hoc* t*-test, where appropriate. Probabilities of <0.05 were considered to be statistically significant. All of the statistical tests were performed with the GraphPad Prism software version 5.0 (GraphPad Software, Inc., San Diego, CA).

## 3. Results

### 3.1. Effect of Berberine and Sirtinol Treatment on Normal Rat Heart

**Figure 2:**
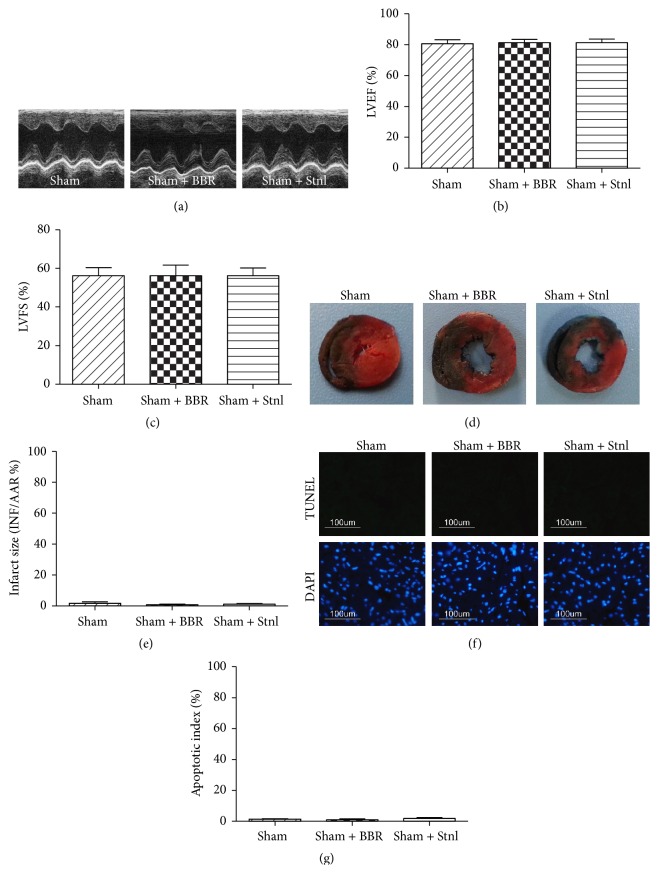
Effects of berberine and sirtinol treatment on normal rat heart. Normal SD rats treated with BBR (200 mg/kg/d for 2 weeks) or Stnl (2 mg/kg/d for 1 week) were subjected to Sham operation. Echocardiographic measurement was carried out after 24 hr of operation. (a) Representative M-mode images by echocardiography. (b) Left ventricular ejection fraction (LVEF). (c) Left ventricular fractional shortening (LVFS). (d) Representative photographs of heart sections. Blue-stained portion indicates nonischemic, normal region; red-stained portion, ischemic/reperfused but not infarcted region; and negative-stained portion, ischemic/reperfused infarcted region. (e) Myocardial infarct size expressed as percentage of area-at-risk (AAR). (f) Representative photomicrographs of in situ detection of apoptotic cardiomyocytes by TUNEL staining. Green fluorescence shows TUNEL-positive nuclei; blue fluorescence shows nuclei of total cardiomyocytes, original magnification ×400. (g) Percentage of TUNEL-positive nuclei. The results are expressed as the mean ± SEM, *n* = 8/group.

**Figure 3:**
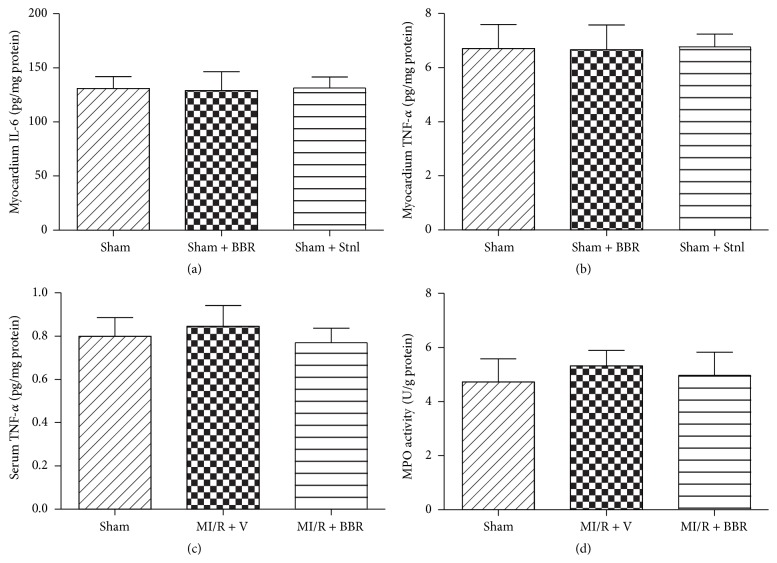
Effects of berberine and sirtinol treatment on inflammation response in normal rat heart. Normal SD rats treated with BBR (200 mg/kg/d for 2 weeks) or Stnl (2 mg/kg/d for 1 week) were subjected to Sham operation. Inflammation markers were measured 6 hr after the surgery. (a) Myocardium IL-6 level. (b) Myocardium TNF-*α* level. (c) Serum TNF-*α* level. (d) Myocardium MPO activity. The results are expressed as the mean ± SEM, *n* = 8/group.

Firstly, we evaluated the effect of BBR and Stnl treatment on Sham operated heart. Under experimental dosages, BBR or Stnl treatment had no significant effect on the LVEF and LVFS compared with the Sham group (*P* > 0.05, Figures 2(a)–2(c)). In addition, myocardial infarct size and apoptotic index were not significantly changed compared with the Sham group (*P* > 0.05, Figures 2(d)–2(g)). Then, we also examined myocardial IL-6 level, myocardium and serum TNF-*α* level, and MPO activity. No significant differences were found among the 3 groups (*P* > 0.05, Figures 3(a)–3(d)).

### 3.2. Effect of Berberine and Sirtinol Treatment on Post-MI/R Cardiac Function

**Figure 4:**
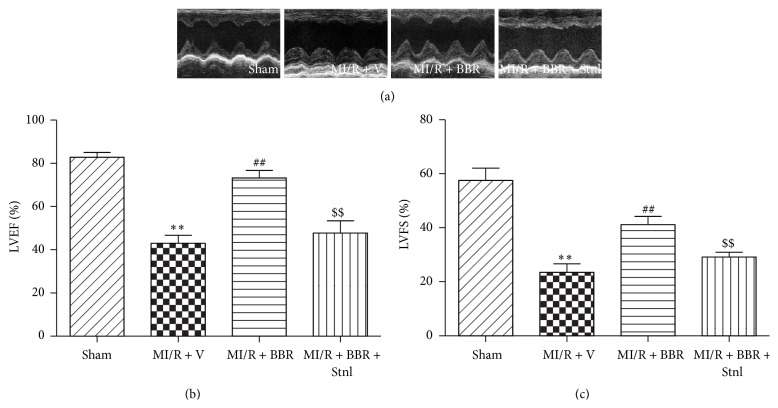
Effects of berberine and sirtinol treatment on post-MI/R cardiac function. Normal SD rats were exposed to BBR treatment (200 mg/kg/d for 2 weeks) in the absence or presence of sirtinol (Stnl, 2 mg/kg/d for 1 week) and then subjected to myocardial ischemia reperfusion (MI/R) operation. Echocardiographic measurement was carried out after 24 hr of reperfusion. (a) Representative M-mode images by echocardiography. (b) Left ventricular ejection fraction (LVEF). (c) Left ventricular fractional shortening (LVFS). The results are expressed as the mean ± SEM, *n* = 8/group. ^*∗∗*^ *P* < 0.01 versus Sham group, ^##^ *P* < 0.01 versus MI/R + V group, and ^$$^ *P* < 0.01 versus MI/R + BBR group.

Compared with the MI/R + V group, BBR treatment significantly improved post-MI/R cardiac function by significantly increased LVEF and LVFS (*P* < 0.01, Figure 4). However, Stnl treatment significantly attenuated BBR's protective effect by decreasing the LVEF and LVFS (*P* < 0.01, compared with the MI/R + BBR group, Figure 4).

### 3.3. Effect of Berberine and Sirtinol Treatment on Apoptotic Index, Infarct Size, Serum LDH, and Serum CK Level in MI/R-Injured Heart

**Figure 5:**
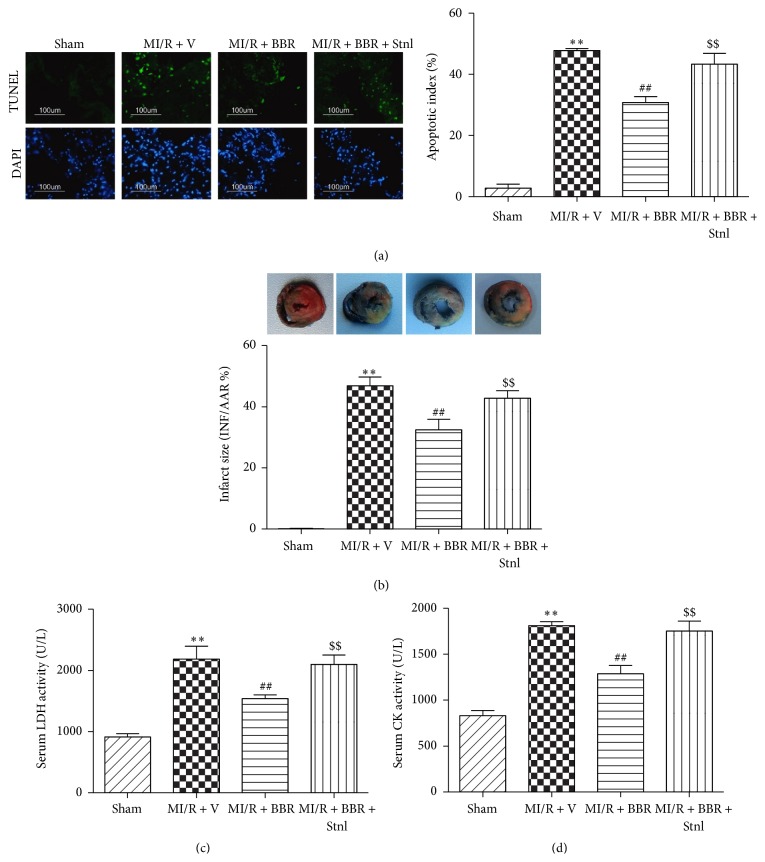
Effects of berberine and sirtinol treatment on apoptotic index, infarct size, serum LDH, and serum CK level in MI/R-injured heart. Normal SD rats were exposed to BBR treatment (200 mg/kg/d for 2 weeks) in the absence or presence of sirtinol (Stnl, 2 mg/kg/d for 1 week) and then subjected to myocardial ischemia reperfusion (MI/R) operation. Apoptotic index, infarct size, serum lactate dehydrogenase (LDH), and serum creatine kinase (CK) level were measured after 6 hr of reperfusion. (a) Left: representative photomicrographs of in situ detection of apoptotic cardiomyocytes by TUNEL staining. Green fluorescence shows TUNEL-positive nuclei; blue fluorescence shows nuclei of total cardiomyocytes, original magnification ×400. Right: percentage of TUNEL-positive nuclei. (b) Top: representative photographs of heart sections. Blue-stained portion indicates nonischemic, normal region; red-stained portion, ischemic/reperfused but not infarcted region; and negative-stained portion, ischemic/reperfused infarcted region. Bottom: myocardial infarct size expressed as percentage of area-at-risk (AAR). (c) Serum LDH level. (d) Serum CK level. The results are expressed as the mean ± SEM, *n* = 8/group. ^*∗∗*^ *P* < 0.01 versus Sham group, ^##^ *P* < 0.01 versus MI/R + V group, and ^$$^ *P* < 0.01 versus MI/R + BBR group.

Next, we found that BBR treatment also significantly decreased the apoptotic index, infarct size, serum LDH, and serum CK levels after 6 hr of reperfusion, which suggested that BBR significantly suppressed MI/RI-induced myocardial apoptosis and necrosis (*P* < 0.01, compared with the MI/R + V group, Figure 5). However, Stnl treatment attenuated these protective effects by significantly increasing apoptotic index, infarct size, serum LDH, and serum CK level (*P* < 0.01, compared with the MI/R + BBR group, Figure 5).

### 3.4. Effect of Berberine and Sirtinol Treatment on Oxidative Stress in MI/R-Injured Heart

**Figure 6:**
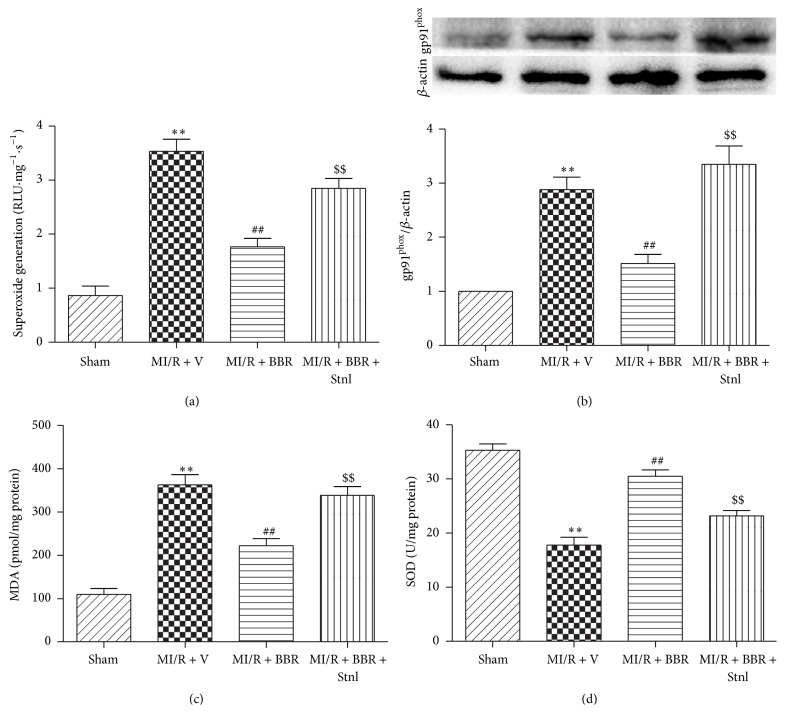
Effects of berberine and sirtinol treatment on oxidative stress in MI/R-injured heart. Normal SD rats were exposed to BBR treatment (200 mg/kg/d for 2 weeks) in the absence or presence of sirtinol (Stnl, 2 mg/kg/d for 1 week) and then subjected to myocardial ischemia reperfusion (MI/R) operation. Cardiac oxidative stress level was measured after 6 hr of reperfusion. (a) Cardiac superoxide generation. (b) gp91^phox^ expression. Top images: representative blots. (c) Myocardial MDA contents. (d) Myocardial SOD contents. The results are expressed as the mean ± SEM, *n* = 8/group. ^*∗∗*^ *P* < 0.01 versus Sham group, ^##^ *P* < 0.01 versus MI/R + V group, and ^$$^ *P* < 0.01 versus MI/R + BBR group.

Furthermore, we measured myocardial superoxide generation, gp91^phox^ protein expression, MDA concentration, and SOD activity. As shown in Figure 6, BBR treatment significantly decreased cardiac superoxide generation as well as MDA concentration, while Stnl significantly attenuated this effect (*P* < 0.01, Figures 6(a) and 6(c)). Afterwards, we determined gp91^phox^ expression, a major component of NADPH oxidase that is the critical superoxide-producing enzyme in the ischemic reperfused heart. As expected, BBR-treated heart showed significantly decreased gp91^phox^ expression compared with the MI/R + V group, while Stnl also suppressed this protective effect (*P* < 0.01, Figure 6(b)). Consistently, significantly reduced levels of MDA concentration were also observed in the MI/R + BBR group compared with the MI/R + V group, while Stnl treatment almost abolished this effect (*P* < 0.01, Figure 6(d)).

### 3.5. Effect of Berberine and Sirtinol Treatment on Inflammation Response in MI/R-Injured Heart

**Figure 7:**
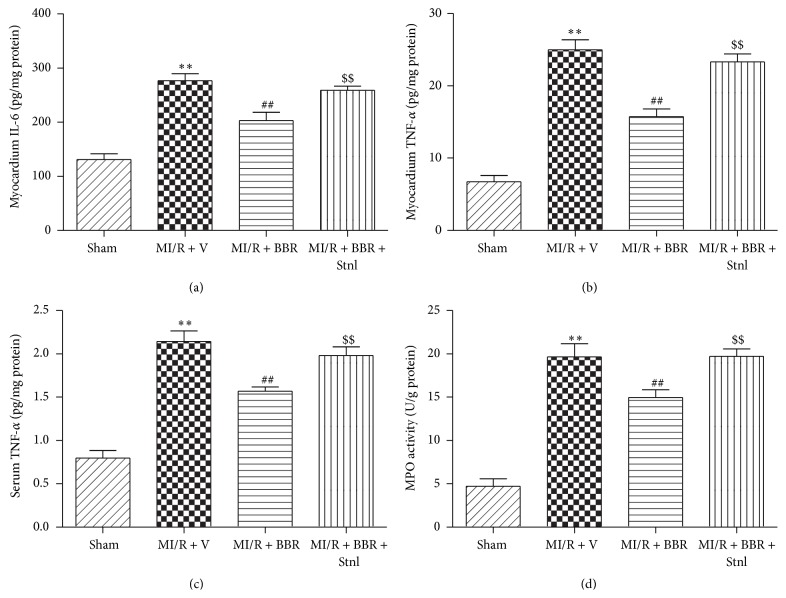
Effects of berberine and sirtinol treatment on inflammation response in normal rat heart. Normal SD rats were exposed to BBR treatment (200 mg/kg/d for 2 weeks) in the absence or presence of sirtinol (Stnl, 2 mg/kg/d for 1 week) and then subjected to myocardial ischemia reperfusion (MI/R) operation. Cardiac oxidative stress level was measured after 6 hr of reperfusion. (a) Myocardium IL-6 level. (b) Myocardium TNF-*α* level. (c) Serum TNF-*α* level. (d) Myocardium MPO activity. The results are expressed as the mean ± SEM, *n* = 8/group. ^*∗∗*^ *P* < 0.01 versus Sham group, ^##^ *P* < 0.01 versus MI/R + V group, and ^$$^ *P* < 0.01 versus MI/R + BBR group.

MI/RI induced large amounts of TNF-*α* in myocardium, which aggravated myocardial damage. We additionally measured myocardial and serum TNF-*α* levels. As seen in Figures 7(b) and 7(c), BBR treatment effectively decreased the levels of TNF-*α* in both myocardium and serum (*P* < 0.01, compared with the MI/R + V group). Moreover, myocardium IL-6 level and MPO activity were also significantly reduced in BBR-treated rats (Figures 7(a) and 7(d), *P* < 0.01, compared with the MI/R + V group).

### 3.6. Effect of Berberine and Sirtinol Treatment on SIRT1, Ac-Foxo1, Caspase-3, Bcl-2, and Bax Expressions in MI/R-Injured Heart

**Figure 8:**
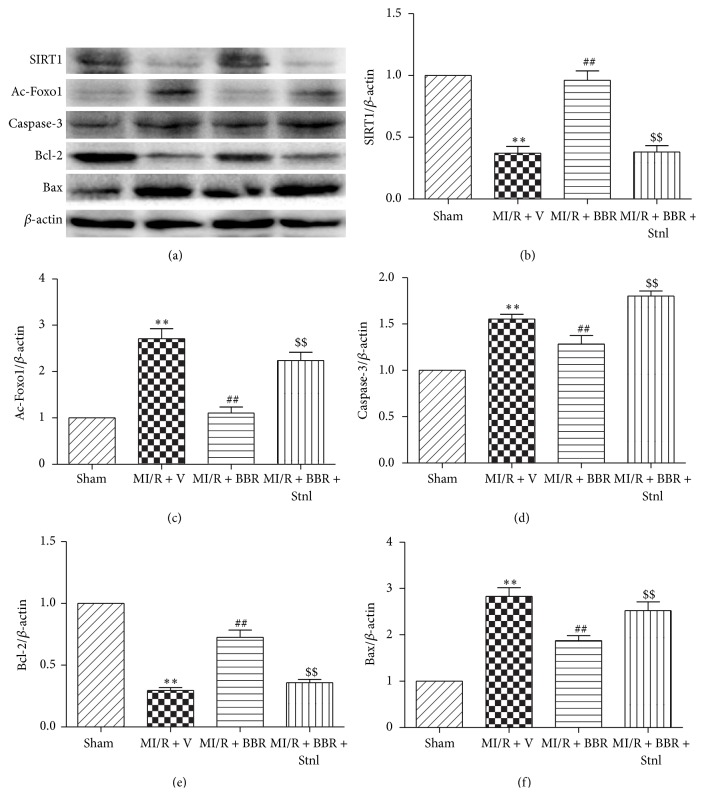
Effects of berberine and sirtinol treatment on SIRT1, Ac-Foxo1, caspase-3, Bcl-2, and Bax expression in MI/R-injured heart. Normal SD rats were exposed to BBR treatment (200 mg/kg/d for 2 weeks) in the absence or presence of sirtinol (Stnl, 2 mg/kg/d for 1 week) and then subjected to myocardial ischemia reperfusion (MI/R) operation. SIRT1-related signaling and apoptosis-related protein were measured after 4 hr of reperfusion. (a) Representative blots. (b) SIRT1 expression. (c) Ac-Foxo1 expression. (d) Caspase-3 expression. (e) Bcl-2 expression. (f) Bax expression. The results are expressed as the mean ± SEM, *n* = 8/group. ^*∗∗*^ *P* < 0.01 versus Sham group, ^##^ *P* < 0.01 versus MI/R + V group, and ^$$^ *P* < 0.01 versus MI/R + BBR group.

Compared with the Sham group, MI/RI significantly decreased SIRT1 expression and increased Ac-Foxo1 expression (*P* < 0.01, Figures 8(b) and 8(c)). Interestingly, BBR treatment significantly upregulated SIRT1 expression and downregulated Ac-Foxo1 expression (*P* < 0.01, compared with the MI/R + V group, Figures 8(b) and 8(c)). However, these effects were almost abolished by Stnl treatment (*P* < 0.01, compared with the MI/R + BBR group, Figures 8(b) and 8(c)). We further measured the apoptotic-related protein expressions. Compared with the MI/R + V group, BBR treatment significantly decreased caspase-3 and Bax expressions and increased Bcl-2 expression (*P* < 0.01, Figures 8(d)–8(f)). As expected, Stnl treatment significantly increased caspase-3 and Bax expressions while decreasing Bcl-2 expression (*P* < 0.01, Figures 8(d)–8(f)).

### 3.7. Effect of Berberine and SIRT1 siRNA on Cell Viability and Apoptotic Index in SIR-Injured Cardiomyocyte

**Figure 9:**
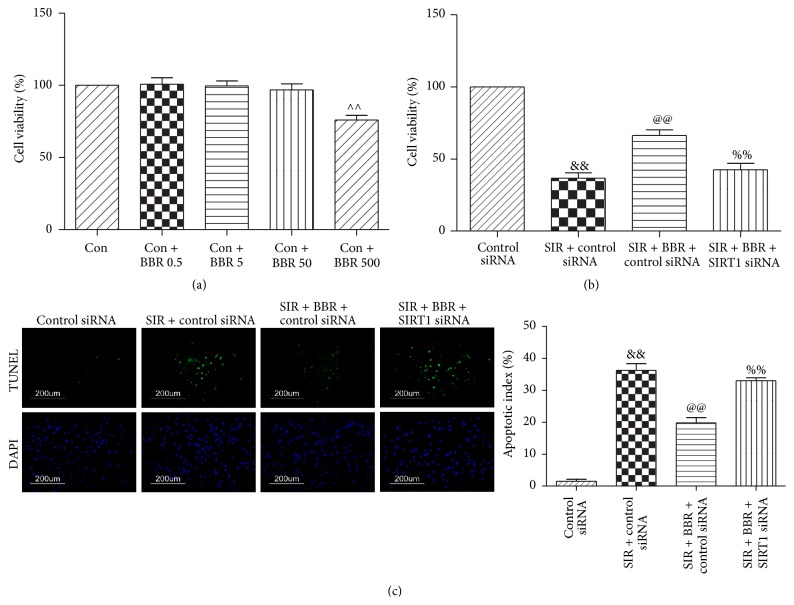
Effects of BBR and SIRT1 siRNA on cell viability and the apoptotic index in SIR-injured cardiomyocyte. H9C2 cardiomyocytes were exposed to control siRNA or SIRT1 siRNA and then treated with BBR (50 *μ*mol/L for 8 hr). Next, cells were subjected to simulated ischemia/reperfusion (SIR, 2 hr/4 hr) treatment. Then, cell viability and apoptotic index were measured. (a) Viability of cardiomyocytes was determined by MTT and was calculated by dividing the optical density of samples by the optical density of Sham control. (b) Viability of cardiomyocytes determined by MTT. (c) Left: representative photomicrographs of in situ detection of apoptotic cardiomyocytes by TUNEL staining. Green fluorescence shows TUNEL-positive nuclei; blue fluorescence shows nuclei of total cardiomyocytes, original magnification ×400. Right: percentage of TUNEL-positive nuclei. The results are expressed as the mean ± SEM, *n* = 8/group. ^∧∧^ *P* < 0.01 versus Con group, ^&&^ *P* < 0.01 versus control siRNA group, ^@@^ *P* < 0.01 versus SIR + control siRNA group, and ^%%^ *P* < 0.01 versus SIR + BBR + control siRNA group.

We treated the cells with BBR at 0.5, 5, 50, and 500 *μ*mol/L for 8 hr and then performed cell viability assay. No significant cell viability changes were found in 0.5, 5, and 50 *μ*mol/L compared with the control group (*P* > 0.05, Figure 9(a)). However, 500 *μ*mol/L BBR treatment significantly affected cell survival (*P* < 0.01, compared with the Con group, Figure 9(a)). Therefore, the dose of 50 *μ*mol/L BBR was chosen in our study. Next, we found that treatment with BBR markedly increased cell viability following SIR injury (*P* < 0.01, compared with the SIR + control siRNA group, Figure 9(b)). Meanwhile, the apoptotic index was also markedly reduced by BBR administration (*P* < 0.01, compared with the SIR + control siRNA group, Figure 9(c)). However, these protective actions were attenuated by SIRT1 siRNA (*P* < 0.01, compared with the SIR + BBR + control siRNA group, Figures 9(b) and 9(c)).

### 3.8. Effect of Berberine and SIRT1 siRNA Treatment on Oxidative Stress and Inflammation Response in SIR-Injured Cardiomyocyte

**Figure 10:**
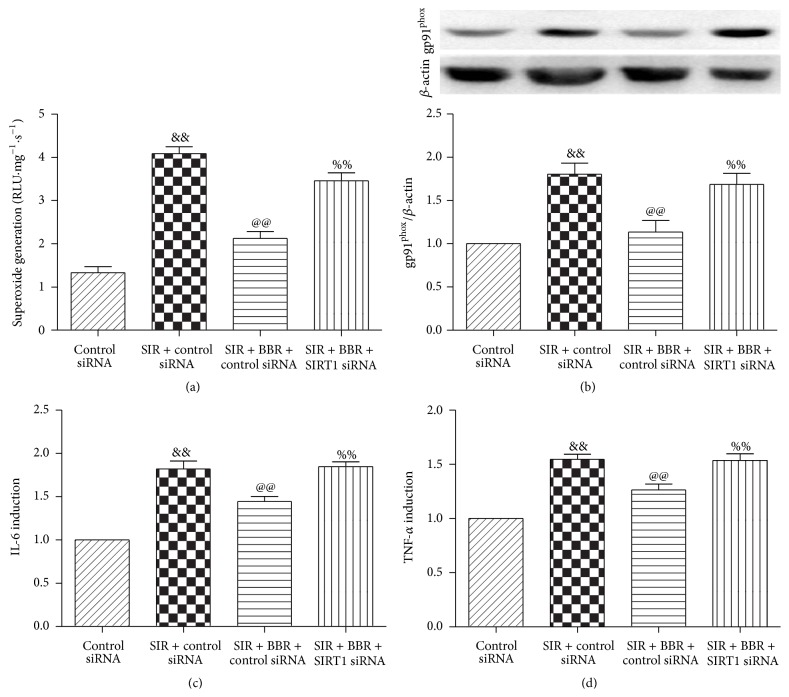
Effects of BBR and SIRT1 siRNA on oxidative stress and inflammation response in SIR-injured cardiomyocyte. H9C2 cardiomyocytes were exposed to control siRNA or SIRT1 siRNA and then treated with BBR (50 *μ*mol/L for 8 hr). Next, cells were subjected to simulated ischemia/reperfusion (SIR, 2 hr/4 hr) treatment. Then, oxidative stress level was measured. (a) Cardiomyocyte superoxide generation. (b) gp91^phox^ expression. Top images: representative blots. (c) Cell supernatants IL-6 level. (d) Cell supernatants TNF-*α* level. The results are expressed as the mean ± SEM, *n* = 8/group. ^&&^ *P* < 0.01 versus control siRNA group, ^@@^ *P* < 0.01 versus SIR + control siRNA group, and ^%%^ *P* < 0.01 versus SIR + BBR + control siRNA group.

We further investigated the oxidative stress level in cultured cardiomyocytes. Compared with the control siRNA group, SIR treatment significantly stimulated ROS production and upregulated gp91^phox^ expression (*P* < 0.01, Figures 10(a) and 10(b)). Consistently, BBR treatment exerted profound antioxidative effect by suppressing ROS production and gp91^phox^ expression (*P* < 0.01, compared with the SIR + control siRNA group, Figures 10(a) and 10(b)). As expected, SIRT1 siRNA treatment attenuated these protective effects (*P* < 0.01, compared with the SIR + BBR + control siRNA group, Figures 10(a) and 10(b)).

Furthermore, we also measured cardiomyocyte inflammation markers in the cultured H9C2 cardiomyocytes. As depicted in Figures 10(c) and 10(d), IL-6 and TNF-*α* levels were both markedly decreased by BBR treatment (*P* < 0.01, compared with the SIR + control siRNA group). As expected, SIRT1 siRNA administration blocked these effects by markedly upregulated IL-6 and TNF-*α* levels, thus aggravating inflammation response induced by SIR damage (*P* < 0.01, compared with the SIR + BBR + control siRNA group, Figures 10(c) and 10(d)).

### 3.9. Effect of Berberine and SIRT1 siRNA Treatment on SIRT1, Ac-Foxo1, Caspase-3, Bcl-2, and Bax Expression in SIR-Injured Cardiomyocyte

**Figure 11:**
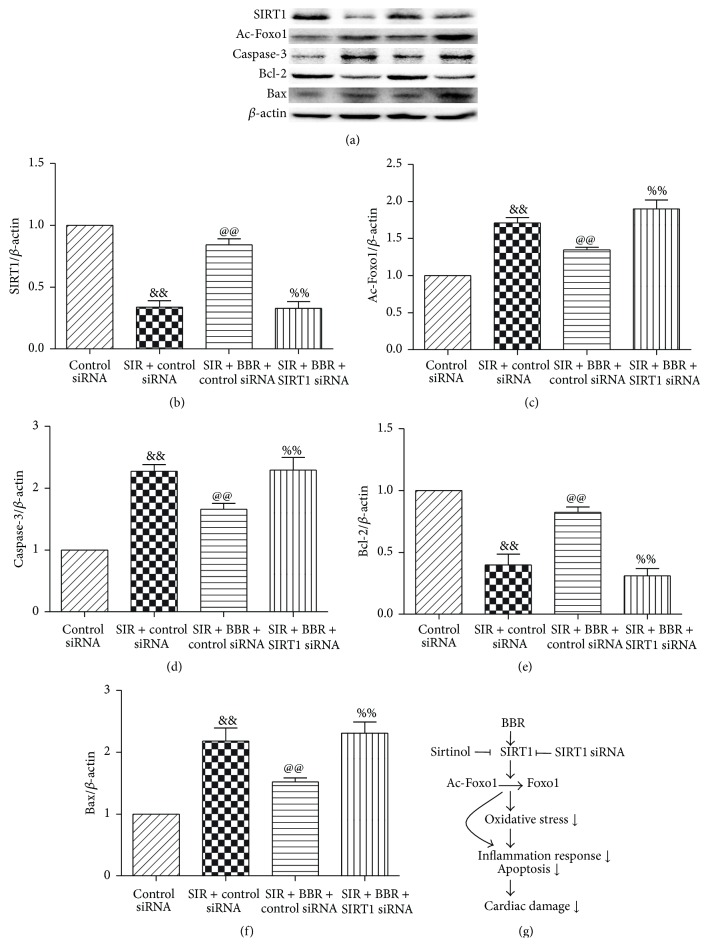
Effects of berberine and SIRT1 siRNA on SIRT1, Ac-Foxo1, caspase-3, Bcl-2, and Bax expression in SIR-injured cardiomyocyte. H9C2 cardiomyocytes were exposed to control siRNA or SIRT1 siRNA and then treated with BBR (50 *μ*mol/L for 8 hr). Next, cells were subjected to simulated ischemia/reperfusion (SIR, 2 hr/4 hr) treatment. Then, SIRT1-related signaling and apoptosis-related protein were measured. (a) Representative blots. (b) SIRT1 expression. (c) Ac-Foxo1 expression. (d) Caspase-3 expression. (e) Bcl-2 expression. (f) Bax expression. (g) Proposed cardioprotective signaling pathway by BBR. The results are expressed as the mean ± SEM, *n* = 8/group. ^&&^ *P* < 0.01 versus control siRNA group, ^@@^ *P* < 0.01 versus SIR + control siRNA group, and ^%%^ *P* < 0.01 versus SIR + BBR + control siRNA group.

Consistent with the in vivo experimental results, BBR treatment significantly increased the expressions of SIRT1 and Bcl-2 and decreased the expressions of Ac-Foxo1, caspase-3, and Bax (*P* < 0.01, compared with the SIR + control siRNA group, Figure 11). However, these effects were significantly inhibited by SIRT1 siRNA treatment (*P* < 0.01, compared with the SIR + BBR + control siRNA group, Figure 11). The proposed cardioprotective signaling pathway by BBR was shown in Figure 11(g).

## 4. Discussion

The major findings from this study are as follows. First, both our in vivo and in vitro studies showed that BBR exerted profound antioxidative and anti-inflammatory effects against MI/R injury. Second, we found BBR's cardioprotective effect might be, at least in part, mediated by SIRT1 signaling. To the best of our knowledge, this is the first report demonstrating the antioxidative and anti-inflammatory actions of BBR and its association with SIRT1 signaling in MI/R condition.

BBR possesses a variety of pharmacological and biological properties and can potentially enhance cardiovascular performance in multiple conditions [29–33]. Attractively, BBR has been demonstrated to reduce MI/R injury [22, 24, 34]. Consistent with these findings, we found that BBR treatment (200 mg/kg/d, 2 weeks) significantly reduced myocardial apoptosis and necrosis, thus improving post-MI/R cardiac functional recovery in rats. Moreover, our in vitro studies also found that BBR incubation (50 *μ*mol/L, 8 hr) effectively inhibited SIR-induced cell death.

Interestingly, previous studies have documented the antioxidative activity of BBR in a number of pathological conditions [11, 13, 35]. However, few studies reported whether BBR could confer similar protective effect against MI/R injury. Importantly, raisanberine, a BBR derivative, was reported to protect pulmonary arterial ring and cardiomyocytes of rat against hypoxia injury by suppressing NADPH oxidase and calcium influx [14]. Qi et al. demonstrated that CPU86017, another BBR derivative, could serve as an effective antioxidant in chronic heart failure rats [15]. Thus, it is rational to hypothesize that BBR might ameliorate oxidative stress induced by MI/RI. In this study, our in vivo and in vitro study provided direct evidence that BBR treatment exerted antioxidative effect by decreasing cardiac superoxide generation, gp91^phox^ expression, and MDA concentration and increasing SOD activity. Notably, MI/R injury also seems to be induced in part by myocardial inflammation response. The underlying mechanisms include (1) cell damage caused by the release of oxygen free radicals and cytotoxic substances, (2) the released inflammatory mediators that cause vascular endothelial cell damage and increased vascular permeability, and (3) further activated inflammatory cells that increase the inflammatory response [36]. Inhibition of myocardial oxidative stress and inflammation response during MI/R injury is of great importance for developing cardioprotective strategies against MI/R [37]. In the present study, we found significantly reduced myocardial IL-6 accumulation and TNF-*α* production in BBR-treated animals or cells, which indicated that BBR also ameliorated cardiac inflammation. We also found markedly reduced myocardial MPO activity in BBR-treated rats. Neutrophil cells contain certain amount of MPO, accounting for 5% of dry cell weight. Hydrogen peroxide reduction can be used to analyze enzyme activity and quantitative determination of the number of neutrophils. Therefore, the activity of MPO in the myocardium of rats was utilized to reflect the infiltration of neutrophils induced by ischemia insult. Taken together, these experimental results showed that BBR possibly attenuated neutrophils infiltration and inflammation response by reducing cardiac oxidative stress during myocardial reperfusion.

SIRT1 is a member of the class III group of histone deacetylases. The deacetylase activity of SIRT1 is dependent on nicotinamide adenine dinucleotide. In MI/R condition, the deacetylase activity of SIRT1 plays a key role in cardioprotection [38]. It has been demonstrated that SIRT1 regulated the acetylation level of Foxo1 to affect apoptotic pathways in the heart. For example, Hsu et al. found that SIRT1 activation protected against MI/R injury by upregulating antioxidants and downregulating proapoptotic molecules through the activation of Foxo1 [39]. We have demonstrated that SIRT1 expression is reduced after MI/RI and activating SIRT1 signaling with curcumin or melatonin confers protective effect against MI/RI by suppressing oxidative stress [22, 38]. Interestingly, several studies have reported that BBR exerted protective action via SIRT1 signaling in some pathological conditions. Gomes and colleagues revealed the critical role for SIRT1 and mitochondrial biogenesis in the preventive effect of BBR on diet-induced insulin resistance [21]. Activating SIRT1 also mediates BBR's hepatoprotective effect against hydrogen peroxide-induced apoptosis [20]. In this study, we found that SIRT1 expression is significantly downregulated after 4 hr of reperfusion, accompanied by increased Ac-Foxo1 expression and enhanced cardiac apoptosis. Both our in vivo and in vitro studies proved that BBR treatment induced a significant increase of SIRT1 expression and Foxo1 deacetylation. The activated SIRT1 signaling was also associated with an increase in the antiapoptotic factor Bcl-2 expression and a decrease in the proapoptotic factor Bax and caspase-3 expressions. As expected, these effects were almost abolished by SIRT1 inhibition. These results all suggest that the cardioprotection of BBR treatment involves the activation of SIRT1 signaling, and BBR-induced SIRT1 activation may decrease the acetylation of Foxo1, thus promoting antiapoptotic signaling.

It has been found that activation of SIRT1 signaling also reduces inflammatory damage [40, 41]. For example, Vinciguerra and colleagues found that SIRT1 signaling mediated the antioxidative and anti-inflammatory actions of the insulin-like growth factor-1 propeptide [42]. In the present study, we found SIRT1 inhibition by sirtinol almost abolished BBR's cardioprotective effects against MI/R injury, indicating that SIRT1 played a key role in BBR's anti-inflammatory action. To further strengthen the mechanisms, we employed SIRT1 siRNA in the in vitro experiment. As expected, SIRT1 siRNA also inhibited BBR's antioxidative and anti-inflammatory effects. Therefore, we conclude that BBR confers protective effect against MI/R injury via SIRT1 signaling, thus alleviating oxidative stress and reducing myocardium inflammation (Figure 11(g)).

In fact, several clinical studies have found exogenous supplementation of BBR reduced cardiovascular risk in patients with moderate hyperlipidemia and excess body weight [43, 44]. Hence, BBR was suggested to be a safe and useful option for the patients in conditions of moderate cardiovascular risk [43, 44]. Based on our data, even short-term consumption of BBR might also be a novel strategy to ameliorate MI/R injury when it is administered several days before cardiac surgery (e.g., open-heart surgery in which extracorporeal circulation is needed). Further clinical investigations are needed before a conclusion can be reached on the utility of BBR in this setting. On the other hand, the present data raised additional questions that we have yet to resolve. In the clinical scenario, the acute cardiac event is mostly unpredictable and we are still waiting for an efficacious molecule that can be administered after myocardial infarction. The cardioprotective effect of BBR treatment after the occurrence of the ischemic event needs to be further studied.

## 5. Conclusion

Our study is the first attempt to investigate the antioxidative and anti-inflammatory effects of BBR and the possible role of SIRT1 signaling in MI/R condition. Our in vivo and in vitro results showed that BBR significantly reduced myocardial damage against ischemia/reperfusion injury possibly due to its strong antioxidant and anti-inflammatory activities. In addition, SIRT1 signaling plays a key role in this condition. These results reveal that BBR may be a promising candidate for the treatment of myocardial ischemia/reperfusion injury in cardiac surgery and ischemic heart diseases.
